# A 10-year follow up of publishing ethics in China: what is new and what is unchanged

**DOI:** 10.1186/s41073-019-0077-3

**Published:** 2019-09-02

**Authors:** Katrina A. Bramstedt, Jun Xu

**Affiliations:** 1 Luxembourg Agency for Research Integrity (LARI), 6, avenue des Hauts-Fourneaux, L-4362 Esch-sur-Alzette, Luxembourg; 2 Bond University Medical Program, University Drive, Gold Coast, Queensland Australia; 3 Department of Vascular Surgery, University of Iowa Hospital and Clinic, 200 Hawkins Drive, Iowa City, IA 52242 USA

**Keywords:** Publishing, Research ethics, Informed consent, China, Research integrity, Organ donation

## Abstract

**Background:**

Organ donation and transplantation in China are ethically complex due to questionable informed consent and the use of prisoners as donors. Publishing works from China can be problematic. The objective of this study was to perform a 10-year follow up on Chinese journals active in donation and transplant publishing regarding the evolution of their publishing guidelines.

**Methods:**

Eleven Chinese journals were analyzed for 7 properties: (1) ethics committee approval; (2) procedure consent; (3) publishing consent; (4) authorship criteria; (5) conflict of interest; (6) duplicate publication; and (7) data integrity. Results were compared with our 2008 study data. Additionally, open access status, impact factor, and MEDLINE-indexing were explored.

**Results:**

Most journals heightened the ethical requirements for publishing, compared to the results of 2008. All 11 now require their published manuscripts to have data integrity. Ten of 11 require ethics committee approval and informed consent for the publication of research studies, whereas in the original study only 2 journals evidenced these requirements. Nine of 11 have criteria for authorship, require conflict of interest disclosure, and forbid duplicate publishing. None of the journals have a policy to exclude data that was obtained from unethical organ donation practices. Nine of 11 journals are MEDLINE-indexed but only 2 are open-access.

**Conclusions:**

Most journals have improved their general ethical publishing requirements but none address unethical organ donation practices.

## Background

*Does a decade change much for transplant publishing ethics in China?* In 2008, the publishing practices of numerous medical journals which are active in the field of solid organ transplantation were explored [1]. Transplantation in China is controversial due to unethical practices such as the use of prisoners as organ donors and lack of informed consent [2]. Scholarly journals which adhere to standard ethical guidelines such as those from the International Committee of Medical Journal Editors [3] forbid publishing of articles which lack ethical assurances such as informed consent and research ethics committee review, but as shown a decade ago [1], many journals from China were willing to publish works lacking these ethical considerations. The current research explores the 11 Chinese journals from the original study, to investigate their current publishing guidelines, in light of more than a decade of continued controversy about questionable Chinese transplant practices, as well as China’s known high rates of plagiarism [4] and duplicate publication [1, 4].

## Methods

Our original study [1] identified 11 journals^1^ from China that had published articles by authors implicated in the Matas-Kilgour investigation of unethical organ donation practices [2]. In December 2018, these 11 journals identified prior were re-analyzed for the same 7 properties: (1) human research studies require approval from the research ethics board; (2) research subjects required to provide informed consent; (3) informed consent required from patients with descriptive or identifying case information; (4) journal specifies authorship criteria; (5) conflict of interest/funding disclosure required; (6) duplicate publication prohibited; (7) article contents must be truthful/data must have integrity. As with our original study, these properties were derived by reading the journal’s website and instructions for authors [JX–pages in Chinese; KB–pages in English and post-translation pages]. Translation from Chinese to English was performed by the co-author of this article [JX]. The results (Table 1) were compared with the data from the 2008 study. Additionally, the impact factor of each journal was searched using the *2018 Edition of the Journal Citation Reports* [5]. The National Library of Medicine online catalog [6] was searched to identify MEDLINE indexing. The journal websites were reviewed for their open-access status.

**Table 1:** Journal Guidelines (2008, 2018)

Journal	Ethics approval	Research informed consent	Publication informed consent	Authorship criteria	COI	No duplicate publishing	Data integrity
*ACTA Academiae Medicinae Sinicae* http://www.actacams.com/CN/column/column32.shtml	No, yes	No, yes	No, yes	No, yes	Yes, no	Yes, no	Yes, yes
*Chinese Critical Care Medicine* http://cmaes.medline.org.cn/Login/tgxz.aspx	U, yes	U, yes	U, no	U, yes	U, yes	U, yes	U, yes
*Chinese Journal of Hepatology* http://cmaes.medline.org.cn/Login/tgxz.aspx	No, yes	No, yes	No, no	No, yes	Yes, yes	Yes, yes	Yes, yes
*Chinese Journal of Integrated* *Traditional and* *Western Medicine***➔** new title: *Chinese Journal of Integrative Medicine* http://www.springer.com/cda/content/document/cda_downloaddocument/2014Instructions+for+Authors.pdf?SGWID=0-0-45-1053737-p161470158	No, yes	No, yes	No, yes	No, yes	No, yes	No, yes	Yes, yes
*Chinese Journal of Surgery* http://cmaes.medline.org.cn/Login/tgxz.aspx	No, yes	No, yes	No, no	Yes, yes	Yes, yes	Yes, yes	No, yes
*Chinese Medical Journal* http://cmaes.medline.org.cn/Login/tgxz.aspx	Yes, yes	Yes, yes	Yes, no	Yes, yes	Yes, yes	Yes, yes	No, yes
*Hepatobiliary and Pancreatic Diseases* *International* https://www.elsevier.com/journals/hepatobiliary-and-pancreatic-diseases-international/1499-3872/guide-for-authors	Yes, yes	Yes, yes	No, yes	Yes, yes	Yes, yes	Yes, yes	No, yes
*Journal of Huazhong University of Science and Technology* *[Medical Sciences]* **➔** new title: *Current Medical Science* http://www.springer.com/cda/content/document/cda_downloaddocument/INSTRUCTIONS++FOR+AUTHORS_11596.pdf?SGWID=0-0-45-445598-p173620803	No, yes	No, yes	No, no	No, no	No, yes	Yes, yes	No, yes
*National Journal of Andrology* http://www.nkxb.cbpt.cnki.net/WKB2/WebPublication/wkTextContent.aspx?navigationContentID=98b47930-5e45-4f40-8d24-47949651169e&mid=nkxb	No, no	No, no	No, no	No, no	No, no	No, no	No, yes
*National Medical Journal of China* *http://cmaes.medline.org.cn/Login/tgxz.aspx*	No, yes	No, yes	No, no	Yes, yes	No, yes	Yes, yes	No, yes
*World Journal of Gastroenterology* ^*†*^ https://www.wjgnet.com/bpg/GerInfo/288	No, yes	No, yes	No, yes	Yes, yes	Yes, yes	No, yes	No, yes

*COI* conflict of interest/funding disclosure required

*U* Unable to obtain instructions for authors from journal in 2008

†Owned by Baishideng Publishing Group Inc. (registered in USA) with its subsidiary, Beijing Baishideng BioMed Scientific Co., Limited (Production Center and Editorial Office) located in China

## Results

As shown in Table 1, all 11 journals now require that manuscript data have integrity (e.g., no fabrication, falsification, deception), whereas in the original study only 3 journals evidenced this requirement. Ten of 11 (90.9%) journals require research ethics committee approval and informed consent for the publication of research studies, whereas in the original study only 2 journals evidenced these requirements. Nine of 11 (81.8%) journals have criteria for authorship, require conflict of interest disclosure, and forbid duplicate publishing practices. Of note, in the original study, *ACTA Academiae Medicinae Sinicae*, required conflict of interest disclosure and forbid duplicate publication; however, their revised author guidelines omit these requirements.

Only 4 of 11 (36.4%) journals require patient consent for the publication of descriptive case studies and/or their associated images. Notably, in the original study, only *Chinese Medical Journal* stated this requirement, but it has been omitted from their current author guidelines.

*National Journal of Andrology* performed consistently poor in terms of publication ethics in both the original study and this re-analysis. In 2008, *National Journal of Andrology* scored 0 of 7 with regard to ethical publishing requirements. In 2018, the journal improved in only one area: they now specifically require manuscript data to have integrity [7]. This journal is MEDLINE-indexed and publishes a variety of works including basic research, clinical research, review articles, clinical experience exchanges, and case reports.

Access to journals can be limited by language and cost. In this study (Table 2), only 2 of 11 (18.2%) journals are published as open-access, providing free availability to scientists and the lay public. The remaining 9 journals allow free access to the article abstracts but limit access to the full-text articles using paywalls. Five of 11 (45.5%) journals publish their articles in English, while the remainder publish their abstracts/summaries and/or tables of contents in English. Nine of 11 (81.8%) journals are currently indexed in MEDLINE, facilitating easy access to their English abstracts.

**Table 2:** Impact factor, indexing, language, access

Journal	Impact factor (2005*, 2018)	MEDLINE-indexed	Language	Open-access or paywall
*ACTA Academiae Medicinae Sinicae*	None, none	Yes	Articles in Chinese; abstracts and table of contents in Chinese and English	Free abstract; paywalled articles
*Chinese Critical Care Medicine*	None, none	No	Articles in Chinese; summaries and table of contents in English	Free abstract; paywalled articles
*Chinese Journal of Hepatology*	None, none	Yes	Articles in Chinese; abstracts and table of contents in Chinese and English	Free abstract; paywalled articles
*Chinese Journal of Integrated Traditional and Western Medicine* **➔** new title: *Chinese Journal of Integrative Medicine*	None, 1.346	Yes	English	Free abstract; paywalled articles
*Chinese Journal of Surgery*	None, none	Yes	Articles in Chinese; Some articles from 2001 to 2002 in English	Free abstract; paywalled articles
*Chinese Medical Journal*	0.561, 1.596	Yes	English	Open access
*Hepatobiliary and Pancreatic Diseases International*	None, 1.500	Yes	English	Free abstract; paywalled articles
*Journal of Huazhong University of Science and Technology [Medical Sciences]***➔** new title: *Current Medical Science*	None, 0.948	No	English	Free abstract; paywalled articles
*National Journal of Andrology*	None, none	Yes	Articles in Chinese; Abstracts and table of contents in Chinese and English	Free abstract; paywalled articles
*National Medical Journal of China*	None, none	Yes	Articles in Chinese; some articles published in English	Free abstract; paywalled articles
*World Journal of Gastroenterology*	3.318, 3.300	Yes	English	Open access

*Reported in Bramstedt and Xu, 2008 (Table 2)

With regard to impact factor, 6 of 11 (54.6%) remain without an impact factor a decade after our initial study. Three of 12 (25%) developed an impact factor as they had none in the prior study. One journal’s impact factor rose greatly (0.561 to 1.596, *Chinese Medical Journal*), while another fell slightly (3.318 to 3.300, *World Journal of Gastroenterology*).

## Discussion

Ease of access (in terms of cost and language) can facilitate the readership of a journal. Specifically, open access [free] and English-language publishing give articles greater visibility, as well as readability. This said journals which are read should have high ethical standards. Additionally, impact factor and indexing are assumed by many to be measures of journal quality. MEDLINE requires their indexed journals to satisfy several “critical elements” including “demonstrating statements indicating adherence to ethical guidelines; evidence that authors have disclosed financial conflicts of interest….” [8]. Journals can also be removed (deselected) from MEDLINE if it discovers major changes in its “scientific quality or editorial process”; [9] however, review cycles for currently indexed journals are not disclosed.

In our study, there has been an improvement in the number of Chinese journals including research ethics and research integrity requirements as a contingency to manuscript submission and acceptance (compared to our initial study in 2008); however, we still found some MEDLINE-indexed journals falling short. Specifically, the distinction between informed consent for research study participation and informed consent for publication of case studies and images (with descriptive or identifying information) is generally not delineated by most journals in our study.

Case studies are generally unique presentations of one or two patients, not part of a committee-approved research study, and thus they lack the committee-approved protocol and consent process. This said publication of such work can potentially pose privacy concerns and should require the consent of the patient if there is descriptive or identifying information with privacy risks. If these case studies involve organ donation and/or transplantation, it is vital that the patient/legal surrogate understand and consent to the procedure. Also, the organ source should always be stated in the manuscript (e.g., living donor, deceased donor, increased risk donor) [10]. Further, all such organs must be ethically sourced [2, 11] and this disclosed in the manuscript submission process [1].

As reported in our original study, these journals were chosen for analysis due to their history of publishing manuscripts about organ donation and transplantation—ethically complex topics (especially in China). It is also important to note that organ donation is an altruistic social good that promotes the goals of medicine, thus is it unfortunate that only 2 of 11 (18.2%) journals in our analysis provide their full-text articles free to scientists, physicians, and the lay public via open access. Knowing that organ donation consistently falls short of need [12] yet is linked to lifesaving transplant technology, it is vital that research and education articles on these topics be readily accessible to physicians, scientists, and society at large, in order to advance science and promote donation.

Since our original work in 2008, other researchers have also called for high ethical publication standards for manuscripts pertaining to organ donation and transplantation [13, 14]. Importantly, some journals such as the *American Journal of Transplantation* [15], *Journal of Clinical Investigation* [16], and *Journal of Heart and Lung Transplantation* [17] refuse to publish articles if the data are derived from an executed prisoner. The journals *Transplantation*, as well as *Transplantation Direct*, both published by The Transplantation Society (www.TTS.org), require that all procedures and studies described “have involved no illegal commercial transactions, the use of organs or other material from executed prisoners, or other unethical practices in obtaining donor organs” [18, 19]. The Transplantation Society is a global professional society of health care workers (e.g., physicians, surgeons, nurses, social workers, ethicists) who work in organ donation and transplant. In this current study of Chinese journals, we found none with a similar explicit ethics requirement on this topic, despite our call for this in our original study [1].

## Conclusions

While Chinese journals avoid addressing the ethical complexity of publishing works pertaining to donation and transplant, they have, in general, responded to our call to heighten their general ethical requirements relating to research ethics committee approval, research informed consent, authorship criteria, conflict of interest, duplicate publishing, and data integrity. Some questions remain: (1) Why are Chinese journals refusing to employ a rule that excludes data sourced from unethical donation and transplant practices? Indeed, such a rule requires journals to have the moral courage to overtly identify and describe unethical donation and transplant practices. This could be difficult if transplant hospitals, procurement services, research funds, and/or publishers are sponsored or supported by the Chinese government, as it has been known to be complicit in such unethical behaviors [11]. While most of the journals in this analysis are not open access, even with low impact factors they are nonetheless not obscure, as 9 of 11 are MEDLINE-indexed, making their content findable and potentially accessible [usable]. This is one of the reasons why excluding unethical data from journals is vital.

Noting that most of the Chinese journals in our study did improve their general publishing ethics requirement, an important question arises: Are these journals upholding their newly adopted general publishing ethics requirements or operating with business as usual? We pose a future study to address this. Lastly, we noted that two journals (*Chinese Medical Journal* and *ACTA Academiae Medicinae Sinicae*) had lowered their publishing standards on some ethical components. Perhaps this was an accidental oversight when they revised their publishing policies as it seems ethically counterproductive to remove requirements of conflict of interest disclosure and informed consent for descriptive/identifying information, as well as forbidding of duplicate publishing. If these requirements are truly eliminated, one wonders if the journal permits publishing that violates these concepts. Our future study will explore this.

## Data Availability

The data that support the findings of this study are available from the authors upon reasonable request.

## Footnotes

1 In the original study, 12 Chinese journals were identified but only 11 were active (one ceased publication).
